# Patient-Reported Outcomes and Experiences Assessment in Women with Breast Cancer: Portuguese Case Study

**DOI:** 10.3390/ijerph20042931

**Published:** 2023-02-08

**Authors:** Anabela Coelho, Candan Kendir, Eliana Barrenho, Niek Klazinga, Cláudia Paiva, Joaquim Abreu de Sousa, Salomé Gonçalves-Monteiro, Patrícia Redondo, Ana Bastos, Armanda Nogueira, Fábio Botelho Guedes, Andreia Silva Costa, Tânia Gaspar

**Affiliations:** 1 Comprehensive Health Research Centre (CHRC), Nursing Department, University of Évora, 7004-516 Évora, Portugal; 2 H&TRC-Health & Technology Research Center, ESTeSL-Escola Superior de Tecnologia da Saúde, Instituto Politécnico de Lisboa, 1549-020 Lisbon, Portugal; 3 Global Health and Tropical Medicine, Instituto de Higiene e Medicina Tropical, Universidade NOVA de Lisboa, 1099-085 Lisbon, Portugal; 4 Organisation for Economic Co-Operation and Development, 75016 Paris, France; 5 Department of Public and Occupational Health, Amsterdam UMC, Amsterdam Public Health Research Institute, University of Amsterdam, 1105 AZ Amsterdam, The Netherlands; 6 Breast Unit, Centro Hospitalar e Universitário do Porto, 4099-001 Porto, Portugal; 7 Department of Surgical Oncology, Portuguese Oncology Institute of Porto (IPO Porto), 4200-072 Porto, Portugal; 8 Porto Comprehensive Cancer Center (Porto.CCC) & RISE@CI-IPOP (Health Research Network), 4200-072 Porto, Portugal; 9 Outcomes Research Lab, Management, Outcomes Research and Economics in Healthcare Group (MOREHealth), Portuguese Oncology Institute of Porto (IPO Porto), 4200-072 Porto, Portugal; 10 Breast Clinic, Portuguese Oncology Institute of Porto (IPO Porto), 4200-072 Porto, Portugal; 11 Quality of Life Office, Portuguese Oncology Institute of Porto (IPO Porto), 4200-072 Porto, Portugal; 12 Instituto de Saúde Ambiental (ISAMB), Faculdade de Medicina, Universidade de Lisboa, 1649-028 Lisbon, Portugal; 13 Nursing Research, Innovation and Development Centre of Lisbon (CIDNUR), Nursing School of Lisbon (ESEL), 1600-096 Lisbon, Portugal; 14 Católica Research Centre for Psychological, Family and Social Wellbeing, Faculdade de Ciências Humanas, Universidade Católica Portuguesa, 1649-023 Lisbon, Portugal; 15 Digital Human-Environment Interaction Labs (HEI-LAB), Universidade Lusófona de Humanidades e Tecnologias, 1749-024 Lisbon, Portugal

**Keywords:** patient-reported outcome measures (PROM), patient reported experience measures (PREM), health system, patient outcomes, breast cancer, public health

## Abstract

In 2020, female breast cancer was the most commonly diagnosed cancer worldwide, representing the type of cancer with the highest incidence among women and the second most common cause of cancer death among women in all OECD countries. The conventional measures addressing the burden of breast cancer by measuring mortality, incidence, and survival do not entirely reflect the quality of life and patients experience when receiving breast cancer care. The main objective of this study is to capture patient-reported outcomes and experiences in women with breast cancer in Portugal using methods developed for international benchmarking purposes, such as the OECD Patient-reported Indicators Surveys. The study included 378 women with breast cancer, with the age distribution being 19.8% aged 15 to 49 years and 80.2% aged 50 years and over. The data collection procedure and analysis followed the “OECD Breast Cancer Patient Reported Outcomes Working Group” protocol, allowing subsequent comparability with data from other OECD member countries. Most women were satisfied with the treatment outcome regarding the shape of their lumpectomy breast when wearing a bra (96.1%) and with the equal size of both breasts (78.3%). Findings on the WHO QOL-BREF showed that women manifest a lower score in well-being when compared with the general population or populations living with chronic diseases. This study shows the feasibility of implementing and using patient-reported metrics (PROM and PREM) in breast cancer services in Portugal. Measuring PROMs and PREMs from Portuguese women receiving breast cancer care provides insightful evidence into the quality and value of cancer care.

## 1. Introduction

Health systems worldwide are working towards maintaining and improving the quality-of-care services by prioritizing patient safety and bringing patients’ voices to promote people-centered healthcare delivery. In order to promote value-based health systems, healthcare providers are compelled to collect data that reflects the real benefits and costs of the care provided that really matter to patients, namely through taking into account the patients’ perspectives through the measurement of patient outcomes including Patient Reported Outcome Measures (PROMs) and their experience of care (Patient Reported Experience Measures, PREMs).

In a volatile, uncertain, complex, and ambiguous environment, such as healthcare, the great benefit of measuring PROMs and PREMs lies in the fact that health facilities, managers, and decision-makers can easily monitor and identify how effective health interventions are in delivering the expected health benefits, both at the individual and/or population level. Moreover, PROMs can be used across different levels of decision-making (macro, meso, and micro) to support macro decisions to define strategic health policies, while helping action at the operational level to ensure good clinical practices defined at the meso level ensure high-quality and safety of care, and informing clinical decision-making at the micro level [1,2]. PROMs promote self-awareness of patients to raise issues with clinicians and promote clinicians’ awareness of patients’ problems [3].

These instruments can measure health in a generic way (using questions that can be used for patients with different conditions in order to compare global health achievements) or in a specific way (using questions regarding a particular disease, condition, or part of the body in order to get the patient’s perceived impact of the disease/condition/intervention on their life) depending on the picture that clinicians/managers and researchers want to have/study [4].

Standardized questionnaires that measure PROMs are increasingly used to give voice to patients and characterize their perception of the impact of the diagnosis, treatment, post-treatment, and survivorship on the quality of their physical, mental, and social health [5,6]. Therefore, the OECD has been calling for the uptake of PROMs and PREMs to complement the conventional outcome measures of mortality and survival rates, in order to measure the aspects of health-related quality of life to assess the real value of health care [7].

These measures provide an opportunity to monitor provider performance, as well as care quality, safety standards, and the impact of the informed policies both at the organizational and system level.

### Condition-Specific PROMS for Breast Cancer

In 2020, female breast cancer was the most commonly diagnosed cancer worldwide, with an estimated 2.3 million new cases [8], representing the type of cancer with the highest incidence among women and the second most common cause of cancer death among women in all OECD countries [9]. Several conventional measures address the burden of breast cancer by measuring mortality, incidence, and survival. However, these indicators do not entirely reflect the patient’s experience of quality-of-life when receiving breast cancer care.

Following a Health Ministerial meeting in 2017, the OECD has assisted countries in adopting systematic measurement of PROMs across different conditions and procedures, particularly breast cancer [10]. For this purpose, an expert group was established in 2018 to advance the standardization, measurement, and use of PROMs in breast cancer care. Currently, the expert group includes over 100 members from more than 20 countries, including international umbrella organizations such as the European Cancer Organisation, patient organizations like EUROPA Donna, and other provider and industry representatives [11]. This has resulted in the OECD delivering two rounds of pilot data collection since 2019 that have been published in the flagship publication Health at a Glance, with some results on breast satisfaction rates following both breast-conserving therapy (BCT) and breast reconstruction [9,12].

BCT is the first surgical option in 60–80% of newly diagnosed cancers of women in Western Europe. The surgical operation removes cancer while preserving the breast tissue as much as possible [13] to achieve the best possible cosmetic outcome. However, changes in the shape, size, and symmetry of the treated breast may occur immediately or years after treatment with unexpected and unintended functional or cosmetic treatment effects for patients [14].

The principal aim of this study is to measure breast surgery outcomes, in particular BCT, reported by Portuguese patients in the first year after treatment.

## 2. Materials and Methods

### 2.1. Study Design

The current study was a prospective transversal study conducted by the research team from the University of Évora and Lusiadas University in Portugal. A total of 10 public hospitals of the National Health Service (NHS) participated in the study. Data was collected, anonymously, from March 2021 to March 2022.

### 2.2. Participants

The study included 378 women with breast cancer that agreed to participate in the postoperative period, with the questionnaires being applied between the 6th and 12th month after breast-conserving surgery.

### 2.3. Instrument

The BREAST-Q scale measurement was the tool chosen to support the data collection following the OECD guidelines for the international data collection on breast cancer PROMs [11]. The BREAST-Q scale was developed and validated to assess the impact and effectiveness of breast surgery to treat breast cancer from the perspective of the patient themselves and is an important measure of the health-related quality of life [15].

The BREAST-Q scale satisfaction with breast following BCT (lumpectomy) module has 11 items over four response hypotheses (1—very dissatisfied; 2—somewhat dissatisfied; 3—somewhat satisfied; 4—very satisfied). These items cover breast appearance in terms of size, symmetry, softness, implant placement, cleavage, and satisfaction with breasts in relation to how a bra fits and how the breasts look when clothed or unclothed. Instructions were given to the participant to fill out the questionnaire as follows:

“The following questions are about your breasts and your breast cancer treatment (by treatment, we mean lumpectomy with or without radiation). If you have had a lumpectomy, answer these questions thinking of the breast you are least satisfied with. With your breasts in mind, in the past, how satisfied or dissatisfied have you been with (…)”.

The translation and back-translation to Portuguese were carried out with the involvement of three researchers and the advice of three medical specialists in the field of breast cancer. The Portuguese version of the instrument showed excellent internal consistency (alpha = 0.93).

To complement data on BREAST-Q, the study also collected data on a complementary variable: the reduced version of the 6-item WHO QOL-BREF scale (WHOQOL) validated by Gaspar (2021) was used to measure the quality of life, with a good internal consistency (alpha = 0.83) [16]. The scale in the present study also shows a good internal consistency (alpha = 0.88).

### 2.4. Procedure

The data collection procedure followed the OECD PaRIS Breast Cancer PROMs Working Group protocol, allowing subsequent comparability with data from other European and OECD countries [11].

Hospitals were invited to participate in the study, awareness-raising meetings were held with the involvement of hospitals, and doubts were clarified with the research team. A total of 10 participating hospitals submitted the project to the respective ethics committees, and the data collection process began after the necessary approvals.

The questionnaire was distributed via an online survey sent by the research team to each participating hospital. Data were collected during a clinical visit by the health care team of each hospital, using the online survey or the paper questionnaire that was thereafter submitted online.

At the end of the data collection process, the research team held an event to present the overall results and drafted a specific report for each of the participating hospitals with conclusions and recommendations.

### 2.5. Inclusion and Exclusion Criteria

The OECD guidelines were followed to define the inclusion and exclusion criteria [11]. According to that, women aged 15 and over were included in the study who received unilateral BCT (lumpectomy). Women that received bilateral or recurrent surgery were excluded.

### 2.6. Data Analysis

A descriptive analysis of frequencies was performed for each of the items of the BREAST-Q and WHO QOL-BREF scale. A mean comparison across groups was performed using the Student’s *t*-analysis using the mean score per global variables (BREAST-Q and WHO QOL-BREF). The study also performed regression analysis under which the WHO QOL-BREF variable was constructed as a continuous variable as it considered all items of the scale.

In order to compare our results with international data we converted the raw scale summed score into a score from 0 (worst) to 100 (best). In this case, higher scores reflected a better outcome.

### 2.7. Ethical Consideration

The study was submitted and approved by all the ethics committees of the participant hospitals as well as by Faculdade de Medicina da Universidade de Lisboa (Reference nº 35/19).

## 3. Results

### 3.1. Population

The study included 378 women with breast cancer, with the age distribution being 19.8% aged between 15 to 49 years and 80.2% aged 50 years and over. Moreover, 10.3% of women reported being smokers; 28.8% had a Body Mass Index (BMI) over 30, indicating obesity; 94.2% reported having had postoperative radiotherapy; and 26.4% had chemotherapy.

### 3.2. Satisfaction with Treatment

Data regarding women’s perceptions of satisfaction with different factors associated with the impact of the intervention on their image and breasts are shown in Table 1. Most women were satisfied with the treatment outcome, varying from 96.1% of women reporting satisfaction with the shape of their lumpectomy breast when wearing a bra to 78.3% of women reporting satisfaction with the equal size of both breasts.

The mean value of satisfaction with treatment total is 3.20, with a standard deviation of 0.69 in a range of 1 to 4 values. However, when we analyze the level of dissatisfaction, we identify that some women revealed greater dissatisfaction with the equality (21.7% dissatisfied) and similarity (18% dissatisfied) of their breasts compared to each other. Women were also more dissatisfied with their image by wearing fitted clothing (17.5% dissatisfied).

In order to compare the results of the present study with international results, the total score value of the BREAST-Q was converted into a scale of 0–100 with the following distribution:

- Total BREAST-Q scored as 80.07 (from 0 to 100);
- Women aged from 15 to 49 years old had a score of 80.20 (from 0 to 100);
- Women aged 50 years old or older had a score of 80.02 (from 0 to 100).

### 3.3. Quality of Life

In relation to the quality of life measured by the WHO QOL-BREF scale, results of this study show a positive perception of quality of life regarding the different indicators detailed in Table 2. Most women (varying from 97.1% concerning the meaning of life to 85.4% in having energy for daily life) were satisfied with their life.

The mean value of WHO QOL-BREF total is 3.75, with a standard deviation of 0.49 in a range of 1 to 5 values.

### 3.4. Comparison of Groups

Comparing group variables such as obesity, radiotherapy and the domains of treatment satisfaction and perceived quality of life (Table 3) showed no statistically significant differences between obese and non-obese women, or between women who did and did not undergo radiotherapy in relation to the WHO QOL-BREF score (*p*-value = 0.330 and 0.640, respectively). However, regarding satisfaction with treatment using the BREAST-Q score, there are statistically significant differences between obese and non-obese women (*p*-value = 0.006) and between women who did and did not undergo radiotherapy (*p*-value = 0.028). Obese women and women who underwent radiotherapy showed less satisfaction with the treatment.

The regression analyses aim to examine the explanatory value of several predictors on women’s satisfaction with their breasts after treatment and their global Quality of Life, namely age, smoking status, obesity, radiation therapy, and chemotherapy, and factors that measure women’s satisfaction. The results of the linear regression analysis presented in Table 4 suggest that factors that most influence quality of life are: “Being able to wear more fitted clothing?” and “How equal in size are your breasts to each other?”. The regression model under study explains 20% (R2 = 0.20) of Quality of Life and factors that measure women’s satisfaction with the impact the treatment has.

## 4. Discussion

PROMs provide information about the outcomes of a given treatment/intervention from the patient’s perspective, including outcomes such as patient satisfaction and quality of life [17].

This study explored the outcomes of breast-cancer surgery in terms of breast satisfaction among Portuguese women using the BREAST-Q score and reported quality of life using the WHO QOL-BREF scale.

**Figure 1:**
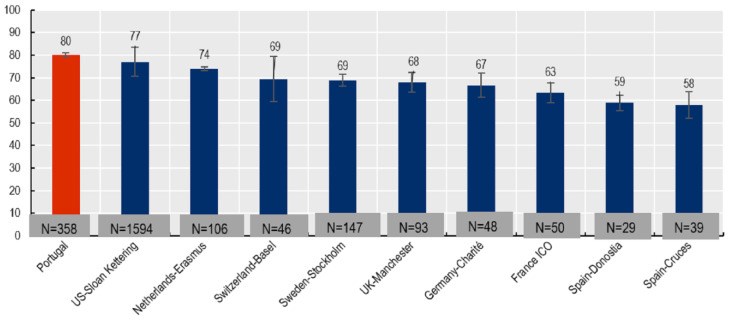
Self-reported breast satisfaction: Crude scores 6–12 months after surgery, 2020–2021. Note: H-lines show 95% confidence intervals. Weighted average based on site sample size was used to calculate crude average breast satisfaction. Source: Data for other countries are retrieved from OECD (2021), Health at a Glance 2021: OECD Indicators, OECD Publishing, Paris, https://doi.org/10.1787/ae3016b9-en (accessed on 12 July 2022).

Different countries worldwide are using PROMs in breast cancer care for improving the quality of care to promote more patient-centered health systems [9], and in Portugal, this strategy seems to reveal that most women are satisfied with their surgical treatment considering that the total BREAST-Q score among our study population was 80.07 (Figure 1), higher than other participating programs of the OECD’s PaRIS initiative with the highest score being 70 [9].

This data is corroborated by another study done in Portugal with 232 patients, that also revealed high satisfaction scores (median 72) with breasts after surgery [18].

High-quality care depends not only on the successful implementation of up-to-date scientific knowledge but also on taking on board the perspective of patients. PROMs were first designed to evaluate clinical trials [19] and applied to clinical practice in order to quantify how effective healthcare interventions are at the individual and/or community level. Standardized and validated questionnaires, such as PROMs and PREMs, measure outcomes and care experience, respectively, making it possible to evaluate healthcare interventions from the patient’s point of view [2].

It is known that the satisfaction level with surgical treatment can be influenced by external factors such as socioeconomic factors, ethnicity, and internal factors related to health literacy and medical knowledge [20], but also by the surgical technique used and the final result (size, shape, and symmetry) [21].

According to our data, a majority of women (96.1%) were satisfied with the shape of their lumpectomy; however, a quarter of the study population was not satisfied with the equality (21.7% dissatisfied) and similarity (18% dissatisfied) of their breasts when comparing to each other, and this can affect their quality of life [22].

This study also showed that women’s perception of their quality of life was explained by some factors including the “equal size” of breasts and the opportunity to wear fitted clothing. Indeed, Waljee (2008) reported that women with pronounced breast asymmetry were significantly more likely to report feeling stigmatized [23] and Dujmović also reported that patients who reported moderate or significant breast asymmetry had worse results on global health status when compared with other patients [24].

The Quality-of-Life score (WHO QOL-BREF) of the women who participated in the study (3.75) was lower than those found in studies with the general population and in the same vein of studies with populations with chronic diseases. In the study conducted by Gaspar et al. (2022) with the general population, the mean value of quality of life was 3.90 [25], which is higher than that of the present study; in the population with chronic disease, the mean value of quality of life was 3.30 [26], which is even lower than that found in the present study.

The findings of this study do not suggest any statistically significant difference in the quality of life reported by women with different background characteristics; nevertheless, studies conclude that surgical management strategies for breast cancer can be influenced by several factors, such as age, lifestyle, and co-morbidities [22], as well as the patient expectation about the functional and cosmetic outcome. In this way, PROMs can be taken as an important tool to assess the self-perception of patients’ health, including their quality of life and body image after treatment, promoting information, communication, and shared decision-making between healthcare professionals and patients [3,5,27,28].

Results of this study show that women are, in general, satisfied with the surgical treatment; however, those who are obese or who received radiotherapy were comparably less satisfied with the treatment, and this is corroborated by several studies that show woman’s postoperative satisfaction can be influenced by age, smoking, obesity, education level, cultural background [20,29], and financial income [29].

In our study, women were satisfied (40.4) or totally satisfied (42.1) with their social relationships. This is an important fact since social relationships can be a major driver for a relatively positive perception of their life. Social relations have been proven important in breast cancer patients [30,31] as well as in other cancers [32,33]. This fact can be used to develop social interventions with patients and their families, aiming to improve women’s quality of life. Psychosocial interventions were already studied previously with good results in what relates to the quality of life [34] and the application of PROMs can improve communication between healthcare professionals and patients, while supporting clinical decision-making, clarifying symptoms, and monitoring disease progression [5].

In the last few decades, several instruments have been developed and tailored to measure clinical practice in the area of breast cancer, with the main challenge being to guarantee the measurement reflects both objective and subjective outcomes [6,35]. Despite several articles suggesting that PROMs improved the quality of life in breast cancer patients, more research is needed to validate a comprehensive measurement of all the factors that affect the quality of life of patients undergoing breast cancer treatment [6].

## 5. Limitations and Suggestions for Future Research and Actions

The study is an important contribution to the understanding of the satisfaction of women with breast cancer regarding their body image after surgery, and the relationship between this satisfaction and their quality of life, which is clearly lower than in the general population. Because it follows OECD guidelines, it allows the comparison of breast satisfaction of Portuguese women with those from other countries and serves as an evidence-basis to inform clinical practice and health policies. Our sample of 10 hospitals may not represent the population as a whole, so results need to be analyzed with caution to avoid general interpretations.

An important aspect to highlight is the fact that this study results from joint work between academia (i.e., two universities and research centers) and 10 NHS hospitals and their teams. This fact demonstrates the value of intersectoral collaboration between academia, healthcare providers, and clinicians, resulting in a more robust engagement with hospital teams, greater knowledge, and empowerment of healthcare professionals in the evaluation and monitoring of the effectiveness of treatments with patients.

It would be important, in future studies, to evaluate the satisfaction with self-image before and after the intervention, and develop a more detailed categorization of the variable “age” into a more detailed range in order to capture the picture of the several stages of life (e.g., adolescence, adult, and elder). The present study does not have this information, but we will collect these specific data in the next survey.

## 6. Conclusions

Measuring PROMs and PREMs from the Portuguese women receiving breast cancer care provides insightful evidence into the quality and value of cancer care. This represents a fundamental step for the continuous improvement of breast cancer care and to promote learning and people-centered health systems.

The results of this study reinforce that patients’ satisfaction and quality of life depend on multiple factors, including pre-existing factors to the treatment (e.g., obesity), conditions related to the treatment (e.g., radiotherapy; breast asymmetry), and other factors unrelated to the treatment (e.g., social relationships).

Policymakers are increasingly recognizing the value of PROMs in many future trends of health systems, such as personalized care, shared decision-making, quality improvement, health systems efficiency, and transparency. Systematic collection of PROMs data will facilitate the transition towards people-centered health systems while improving the quality of care and clinical decision-making.

## Figures and Tables

**Table 1:** Descriptive statistical analysis of treatment satisfaction (BREAST-Q).

BREAST-Q Questions	1 Very Dissatisfied (%)	2 Somewhat Dissatisfied (%)	3 Somewhat Satisfied (%)	4 Very Satisfied (%)
How you look in the mirror clothed?	1.6	3.7	58.2	36.5
The shape of your lumpectomy breast when you are wearing a bra?	0.5	3.4	57.5	38.6
How normal you feel in your clothes?	1.1	3.2	55.5	40.2
Being able to wear clothing that is more fitted?	2.9	14.6	52.1	30.4
How your lumpectomy breast sits/hangs?	0.0	6.1	61.1	32.8
How smoothly shaped your lumpectomy breast looks?	0.8	8.7	53.5	37.0
The contour (outline) of your lumpectomy breast?	0.5	7.4	56.9	35.2
How equal in size your breasts are to each other?	2.1	19.6	54.2	24.1
How normal your lumpectomy breast looks?	0.3	12.2	57.9	29.6
How much your breasts look the same?	1.9	16.1	56.1	25.9
How you look in the mirror unclothed?	1.3	12.4	60.8	25.4

**Table 2:** Descriptive statistical analysis of Quality of Life (WHO QOL-BREF).

WHO QOL-BREF	1 Totally Unsatisfied (%)	2 Not Very Satisfied (%)	3 Moderately Satisfied (%)	4 Well Satisfied (%)	5 Totally Satisfied (%)
*Satisfied with your health*	0.8	7.9	46.0	37.0	8.2
*Life has meaning*	0.3	2.6	23.8	42.6	30.7
*Energy for your daily life*	0.8	13.8	38.6	29.9	16.9
*Ability to perform everyday activities*	1.6	11.4	39.7	31.0	16.4
*Self-satisfaction*	0.8	5.6	24.3	44.7	24.6
*Satisfied with social relationships (friends, family, colleagues, etc.)*	0.8	2.4	14.6	40.2	42.1

**Table 3:** Descriptive Statistics and Comparison analysis according to BMI and radiotherapy.

	Descriptive Statistics				*t*-Test & Significance
	χ	*SD*	χ	*SD*	
	BMI ≥ 30		BMI < 30		
Treatment satisfaction (BREAST-Q)	3.10	0.46	3.25	0.50	*t* = −2.69, *p* = 0.006
Quality of life (WHO QOL-BREF)	3.67	0.70	3.78	0.69	*t* = −1.51, *p* = 0.330 (n.s.)
	Radiotherapy		No radiotherapy		
Treatment satisfaction (BREAST-Q)	3.19	0.50	3.43	0.39	*t* = −2.20, *p* = 0.028
Quality of life (WHO QOL-BREF)	3.75	0.68	3.68	0.81	*t* = 0.47, *p* = 0.640 (n.s.)

**Table 4:** Linear regression model to study Quality of Life.

	B	Error	*Β*	T	*p*
(Constant)	2.680	0.508		5.273	0.000
Age group	0.008	0.102	0.005	0.076	0.939
Smoking status (1 = yes)	−0.134	0.130	−0.065	−1.031	0.304
Obesity (1 = yes)	0.058	0.100	0.036	0.582	0.561
Radiotherapy (1 = yes)	−0.016	0.158	−0.006	−0.101	0.920
chemotherapy (1 = yes)	−0.102	0.092	−0.068	−1.107	0.270
How you look in the mirror clothed?	0.213	0.204	0.073	1.046	0.297
The shape of your lumpectomy breast when you are wearing a bra?	0.259	0.279	0.074	0.927	0.355
How normal you feel in your clothes?	0.306	0.315	0.072	0.973	0.332
Being able to wear clothing that is more fitted?	0.265	0.129	0.139	2.050	0.042
How your lumpectomy breast sits/hangs?	−0.133	0.251	−0.040	−0.528	0.598
How smoothly shaped your lumpectomy breast looks?	−0.118	0.225	−0.055	−0.524	0.601
The contour (outline) of your lumpectomy breast?	0.263	0.230	0.107	1.145	0.253
How equal in size your breasts are to each other?	0.402	0.194	0.239	2.074	0.039
How normal your lumpectomy breast looks?	0.289	0.188	0.144	1.539	0.125
How much your breasts look the same?	−0.251	0.228	−0.135	−1.099	0.273
How you look in the mirror unclothed?	0.071	0.165	0.035	0.429	0.668

R2 = 0.20 F = 3.53 (16/222), *p* < 0.001. Dependent variable = Quality of Life (WHO QOL-BREF).

## Data Availability

The data presented in this study are available on request from the corresponding author. The data are not publicly available due to ownership belonging to the institutions where the study was conducted.
